# Supplementary figures and images for: MCT1-dependent energetic failure and neuroinflammation underlie optic nerve degeneration in Wolfram syndrome mice
Source: eLife. 2023 Jan 16;12:e81779. doi: 10.7554/eLife.81779 (PMC9891717; doi:10.7554/eLife.81779)

**Figure 5A**

**STAT3**

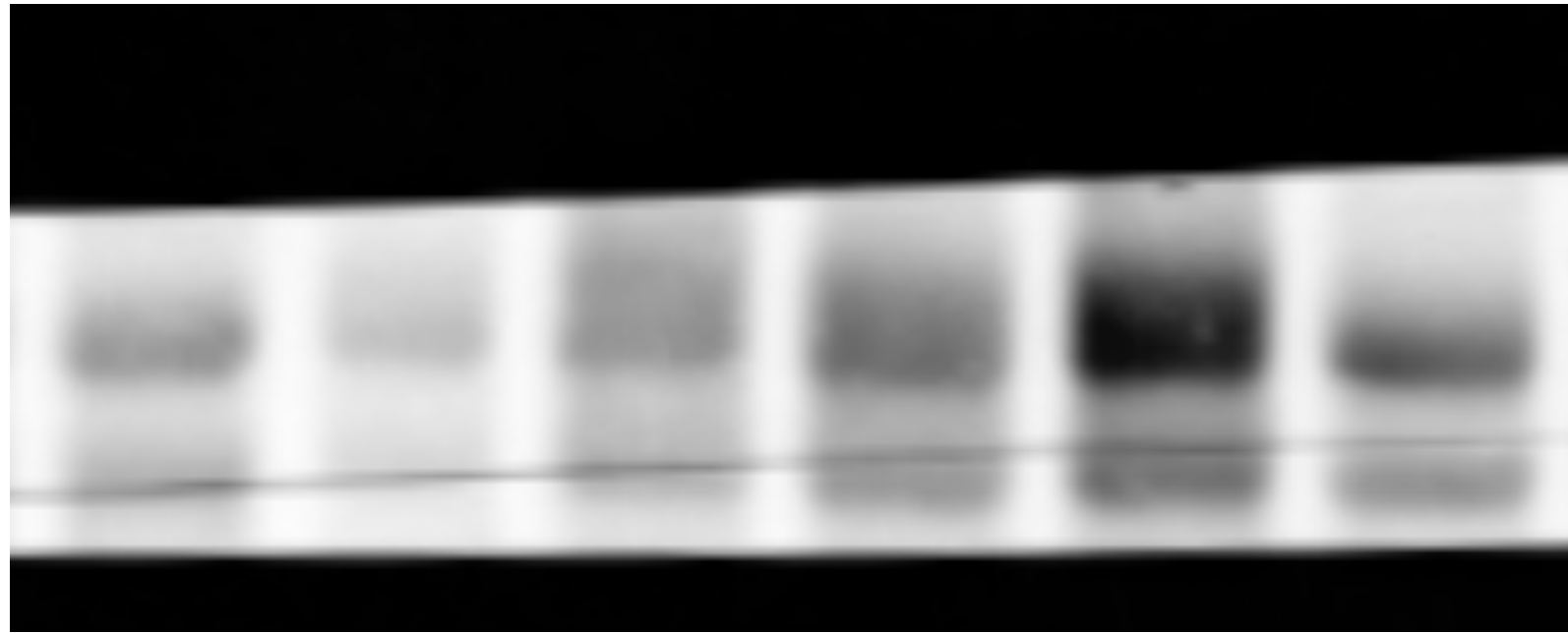

**ACTIN**

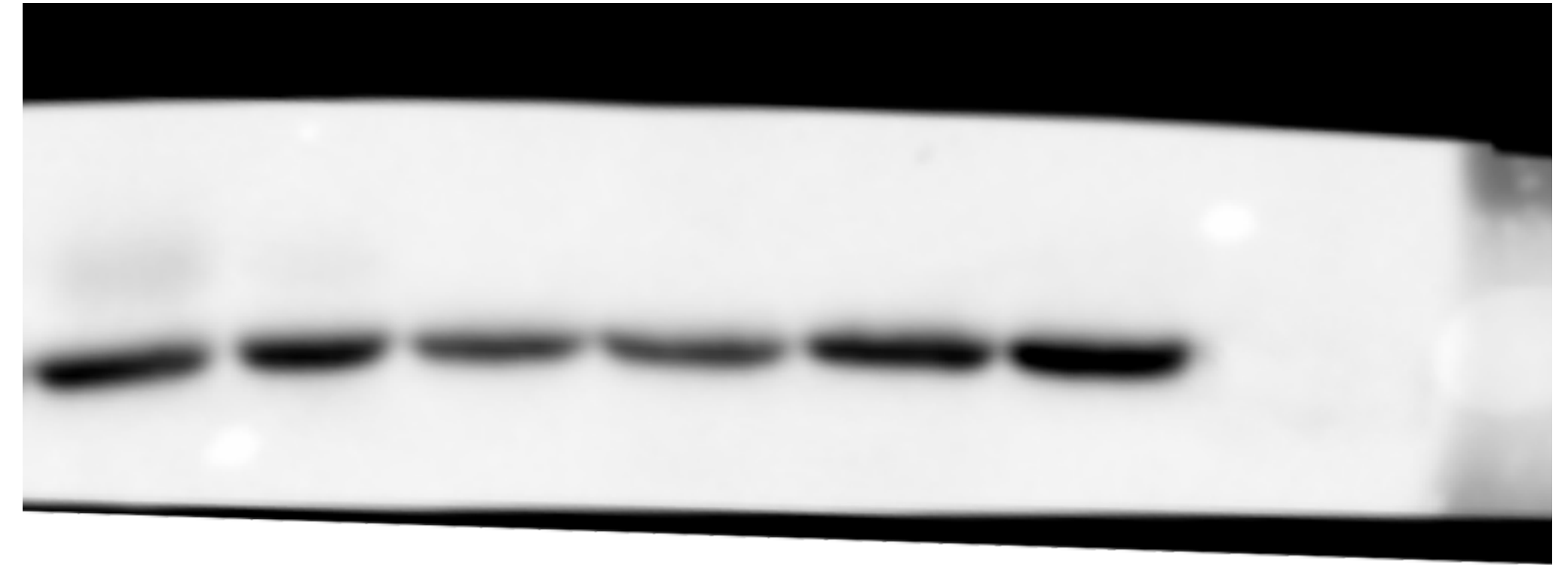

**p-STAT3**

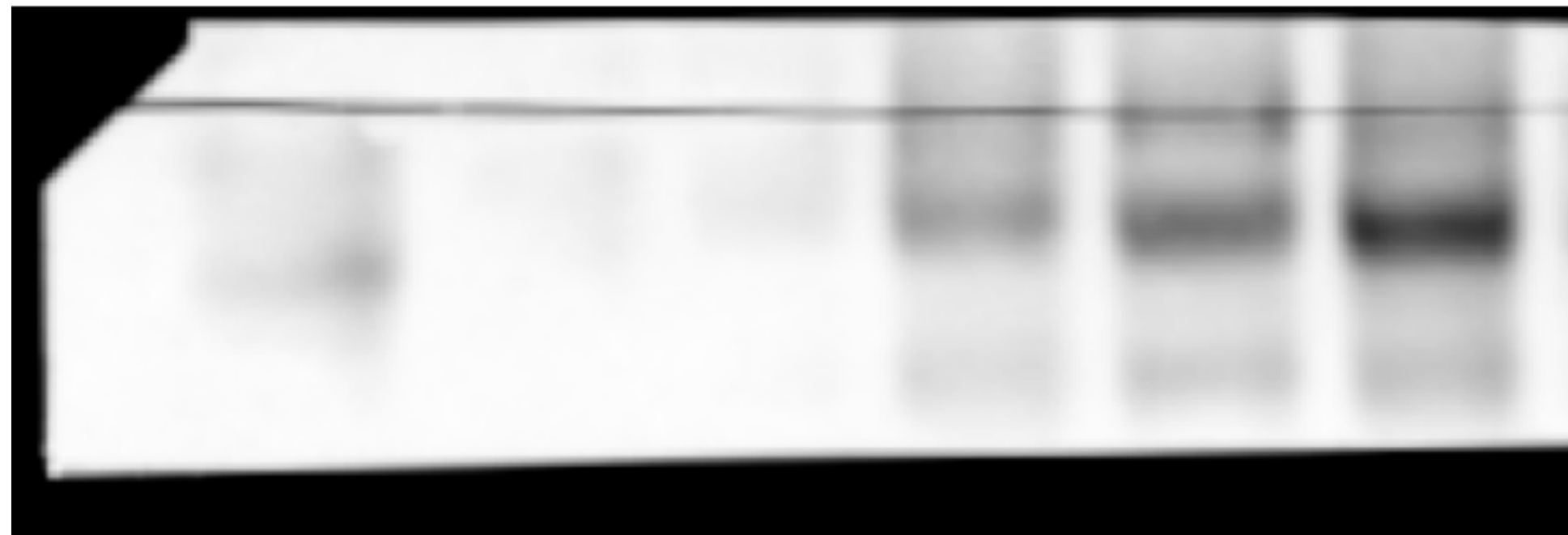

**ACTIN**

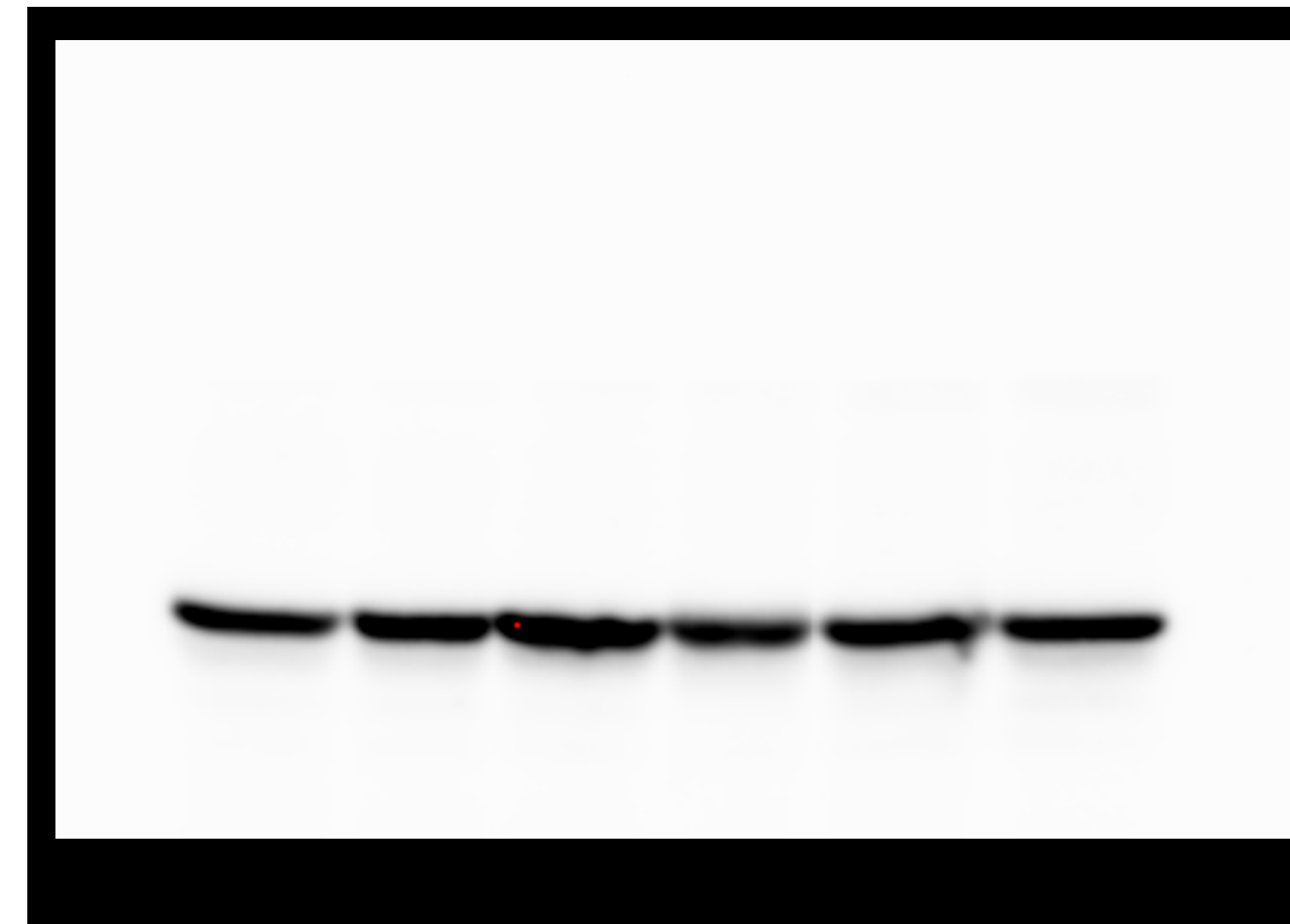

Supplement: Figure 5—source data 1. [file elife-81779-fig5-data1.pdf]

**Figure 6C**

**WFS1**

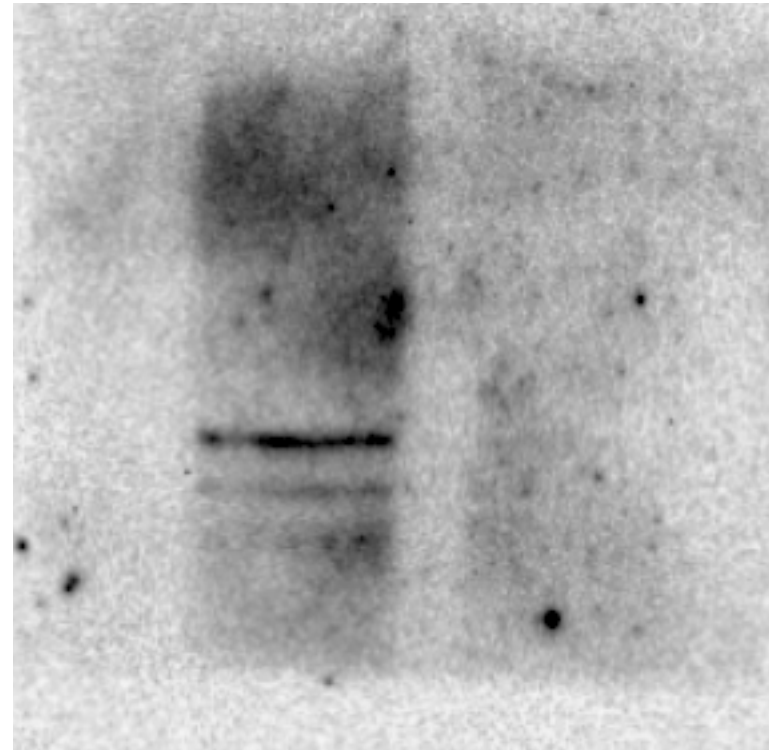

**MCT1**

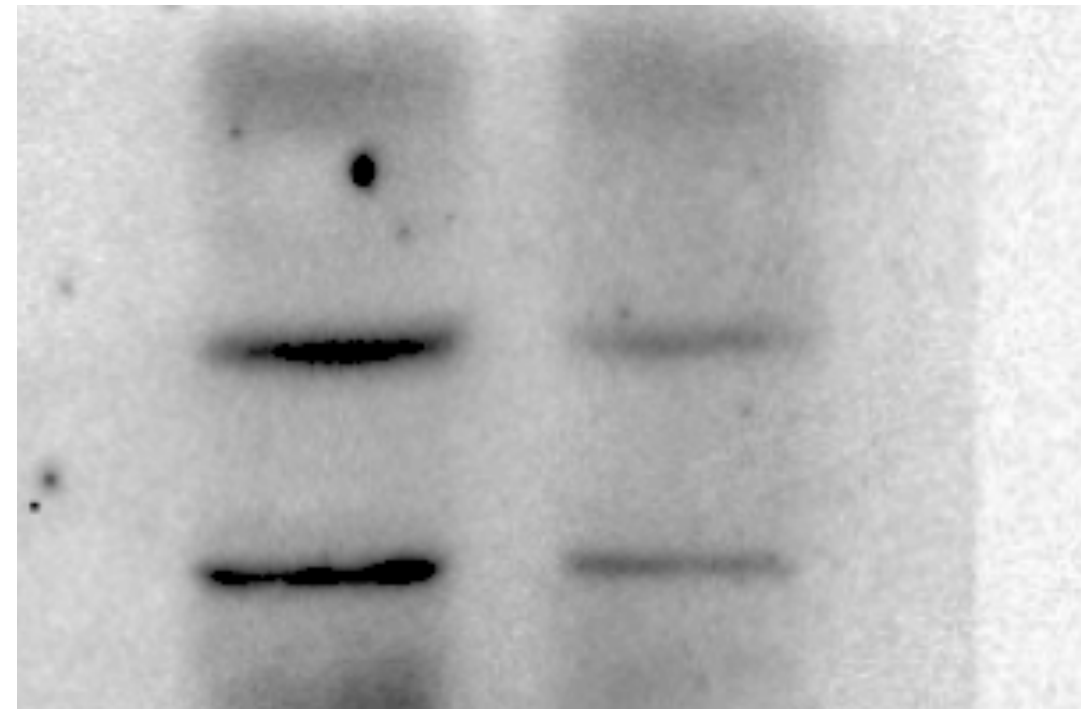

**Basigin**

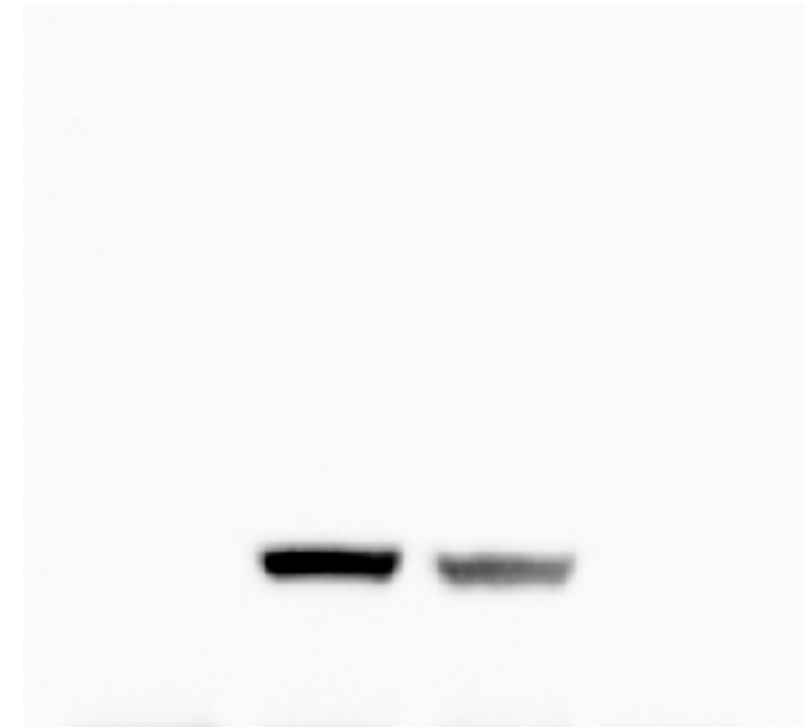

**CLNX**

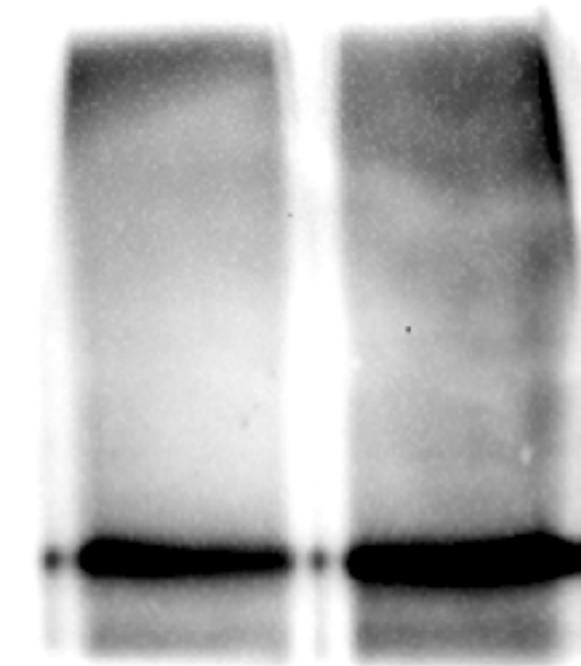

**WFS1**

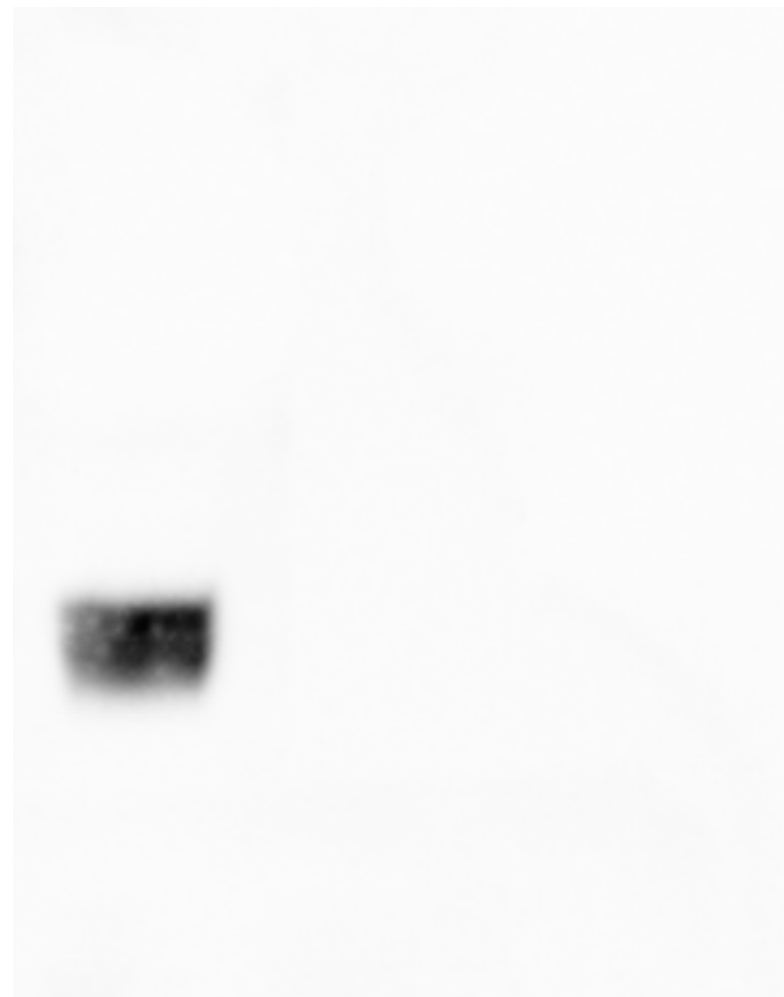

**MCT1**

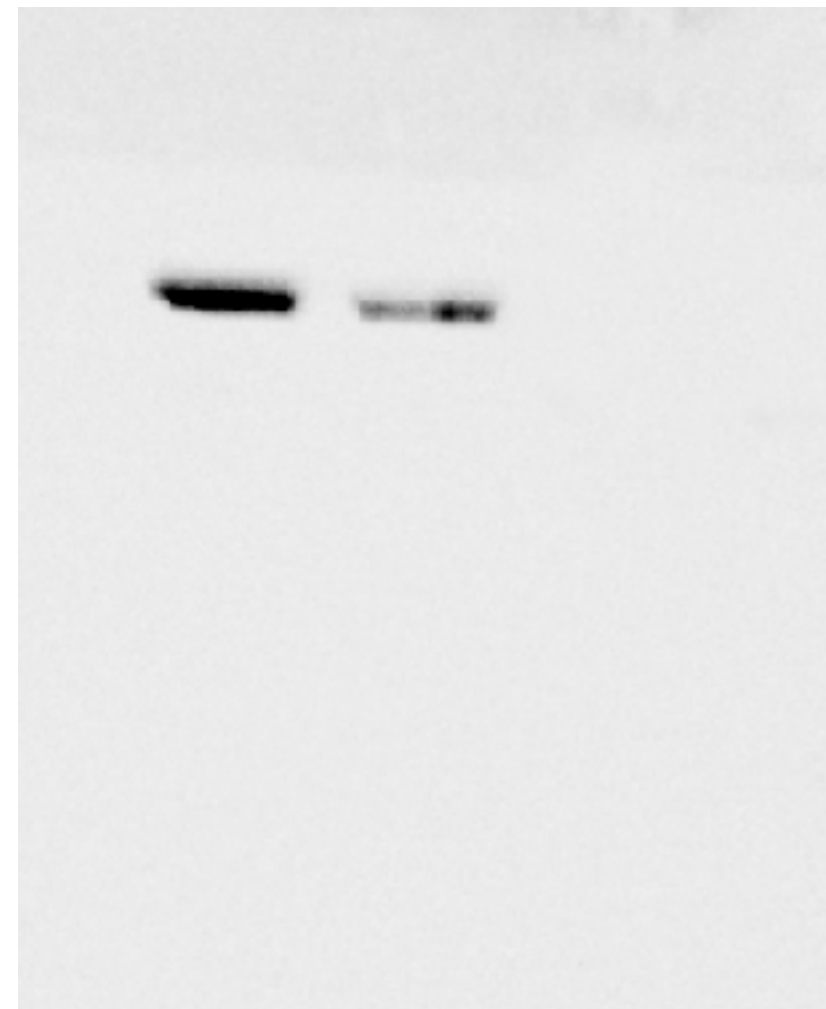

**MCT2**

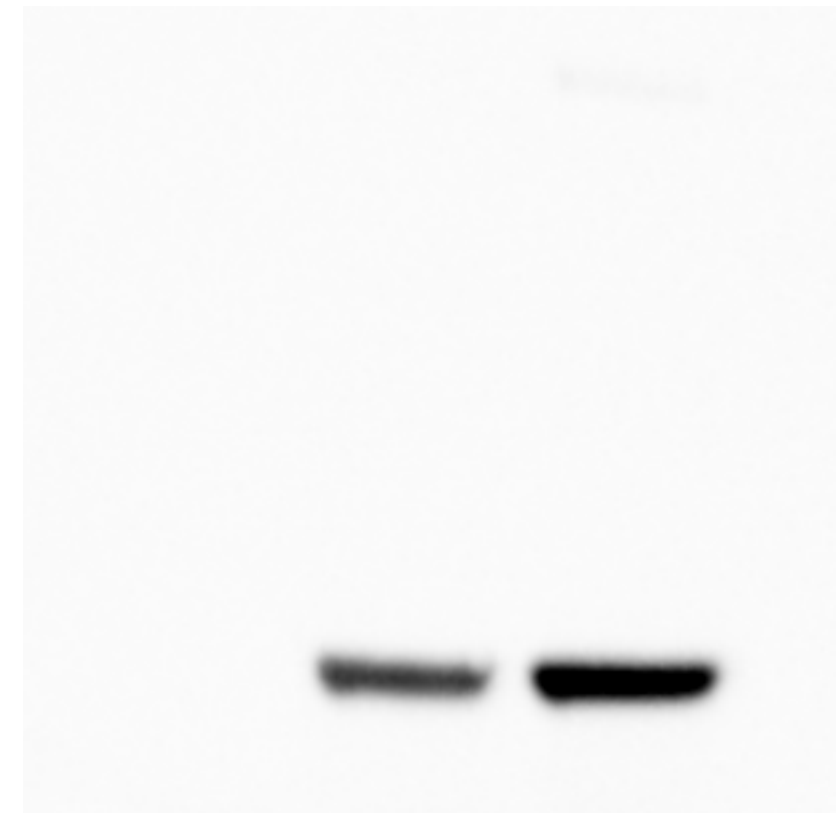

**CLNX**

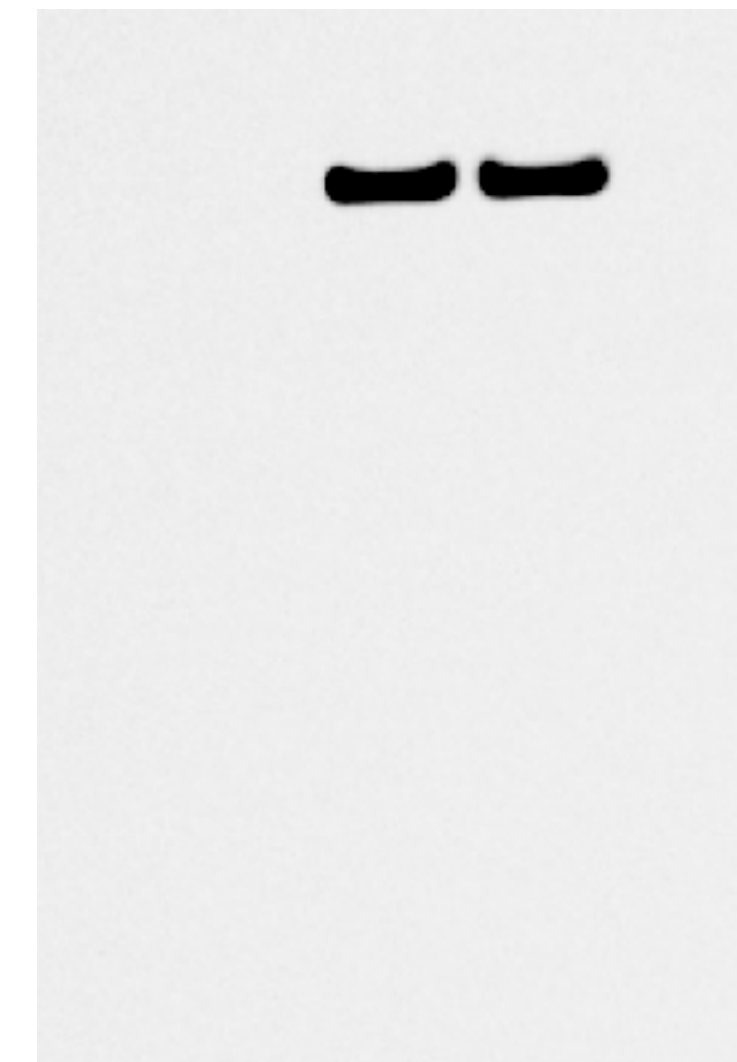

Supplement: Figure 6—source data 1. [file elife-81779-fig6-data1.pdf]

**Figure 6D**

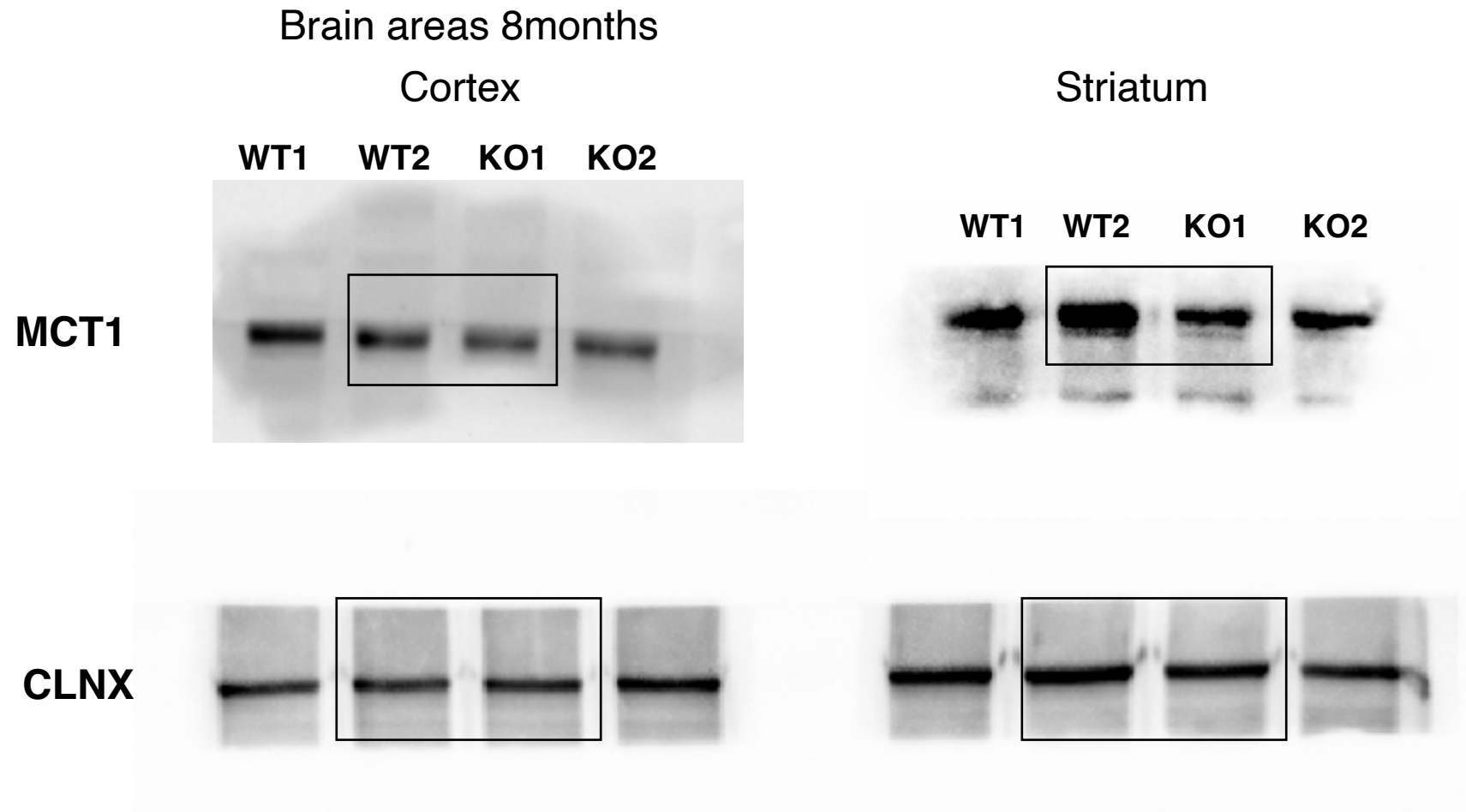

Supplement: Figure 6—source data 2. [file elife-81779-fig6-data2.pdf]

**Figure 6E**

**RFP-Input**

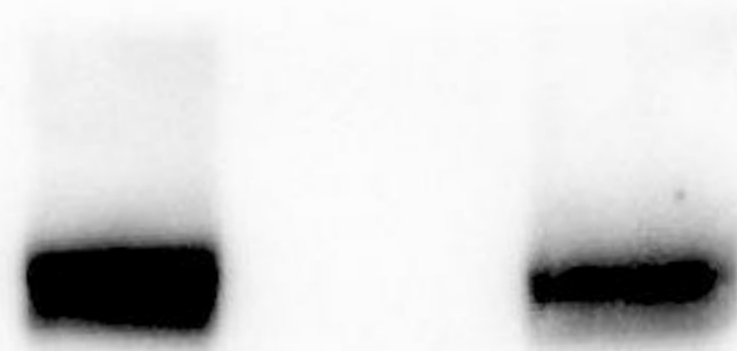

**V5-Input**

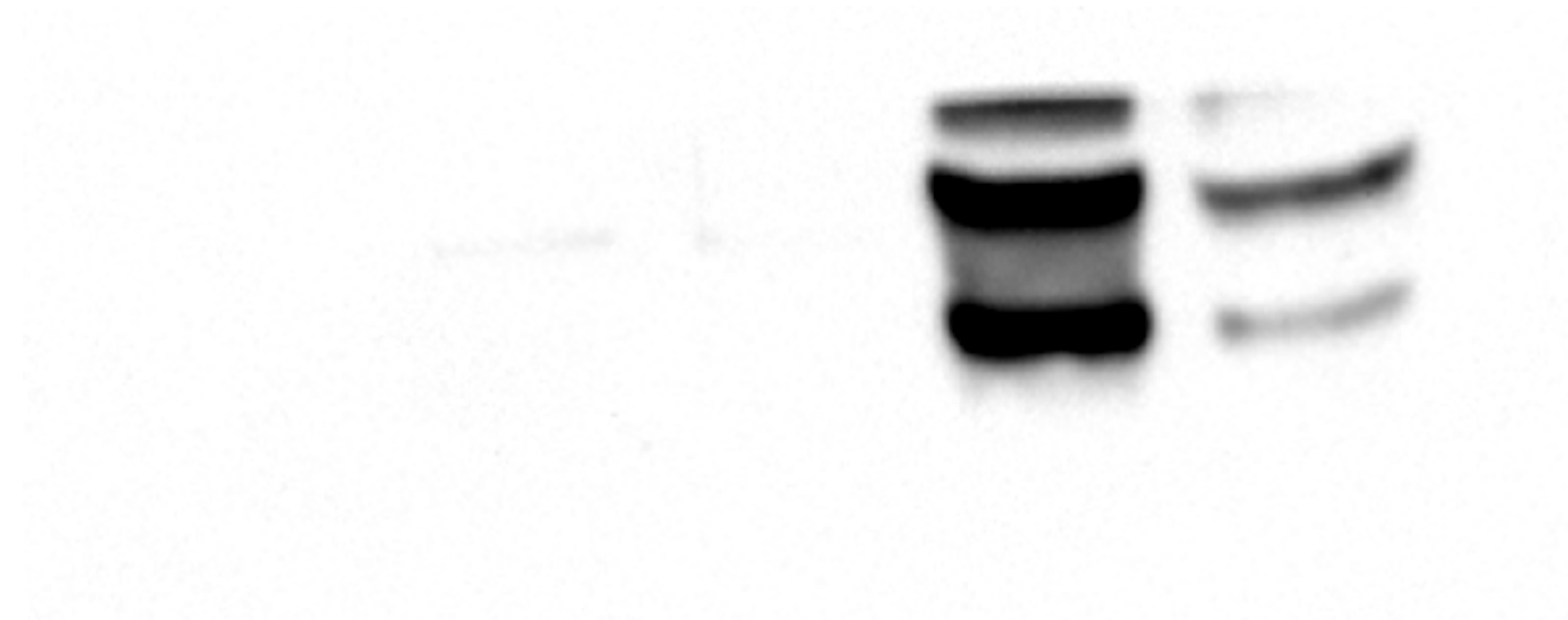

**RFP-IP**

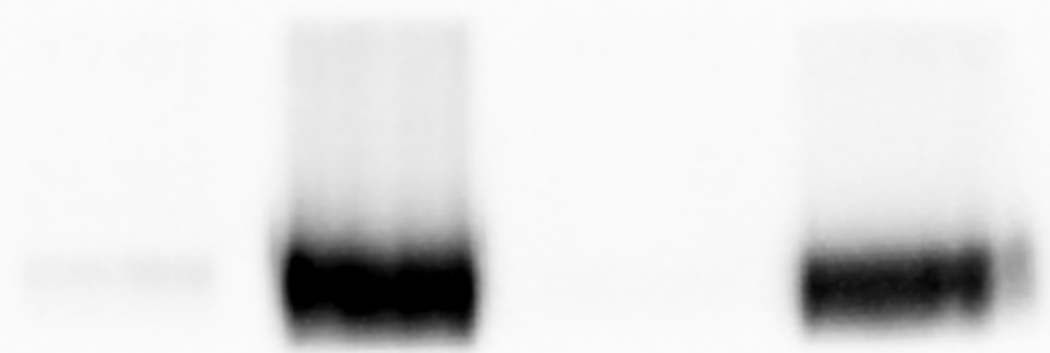

**RFP-IP**

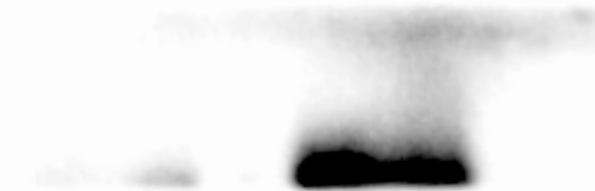

Supplement: Figure 6—source data 3. [file elife-81779-fig6-data3.pdf]

## Figure 6-supplemental figure 2

### MCT1

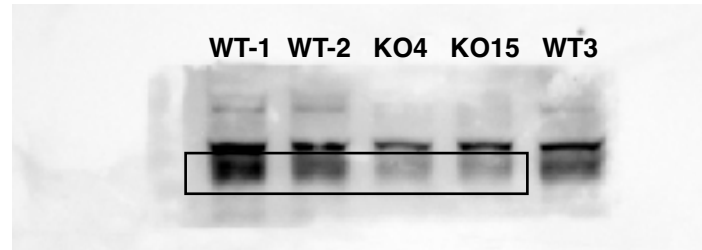

### WFS1

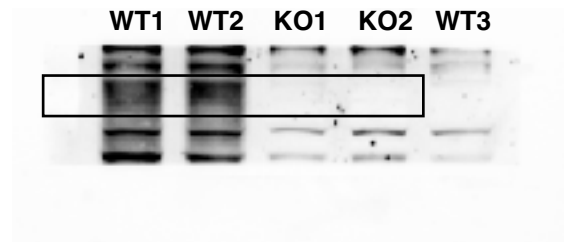

### CLNX

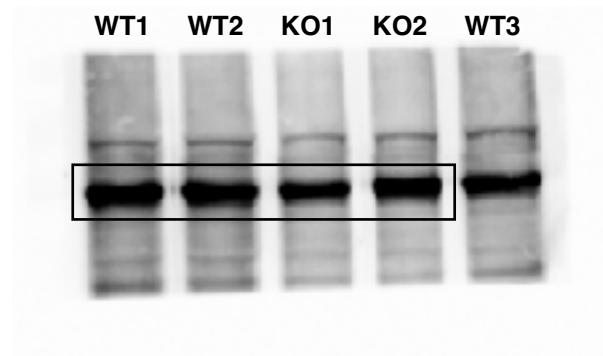

Supplement: Figure 6—figure supplement 2—source data 1. [file elife-81779-fig6-figsupp2-data1.pdf]

**Figure 7B**

**WFS1**

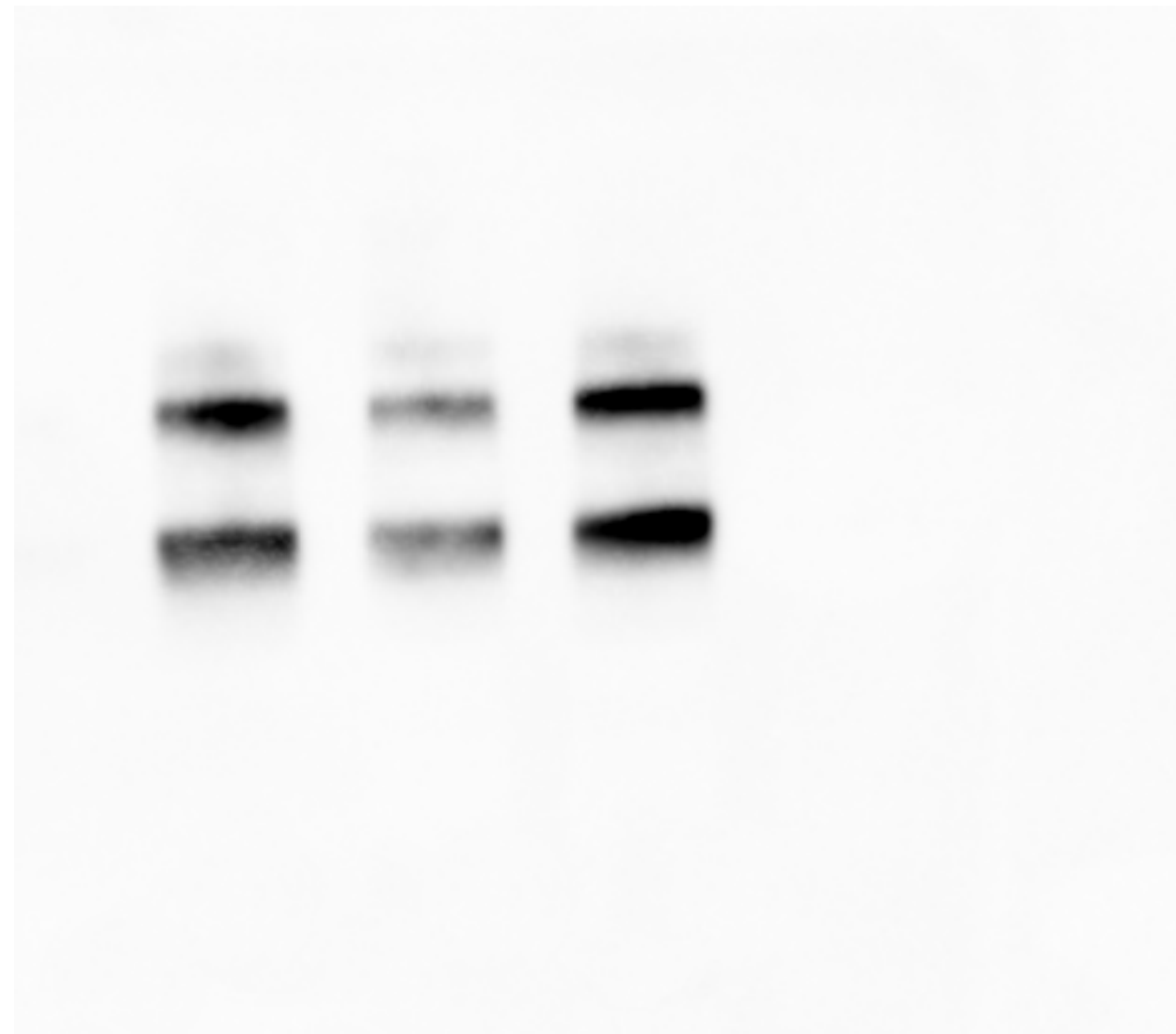

**MCT1**

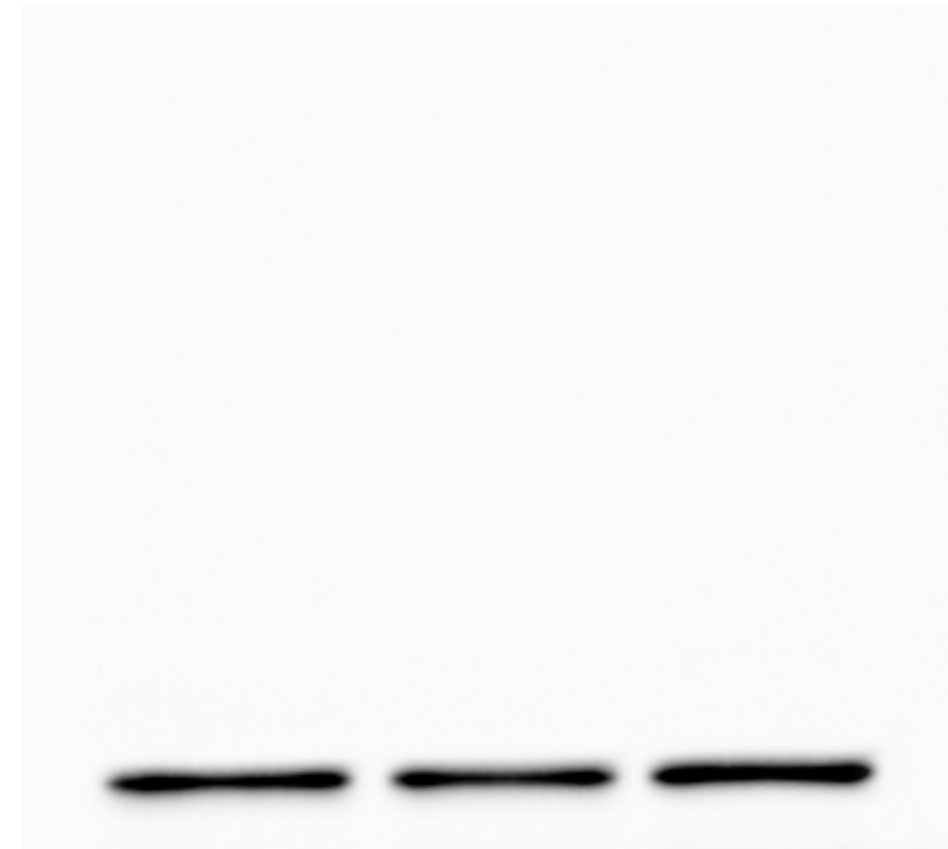

**ACTIN**

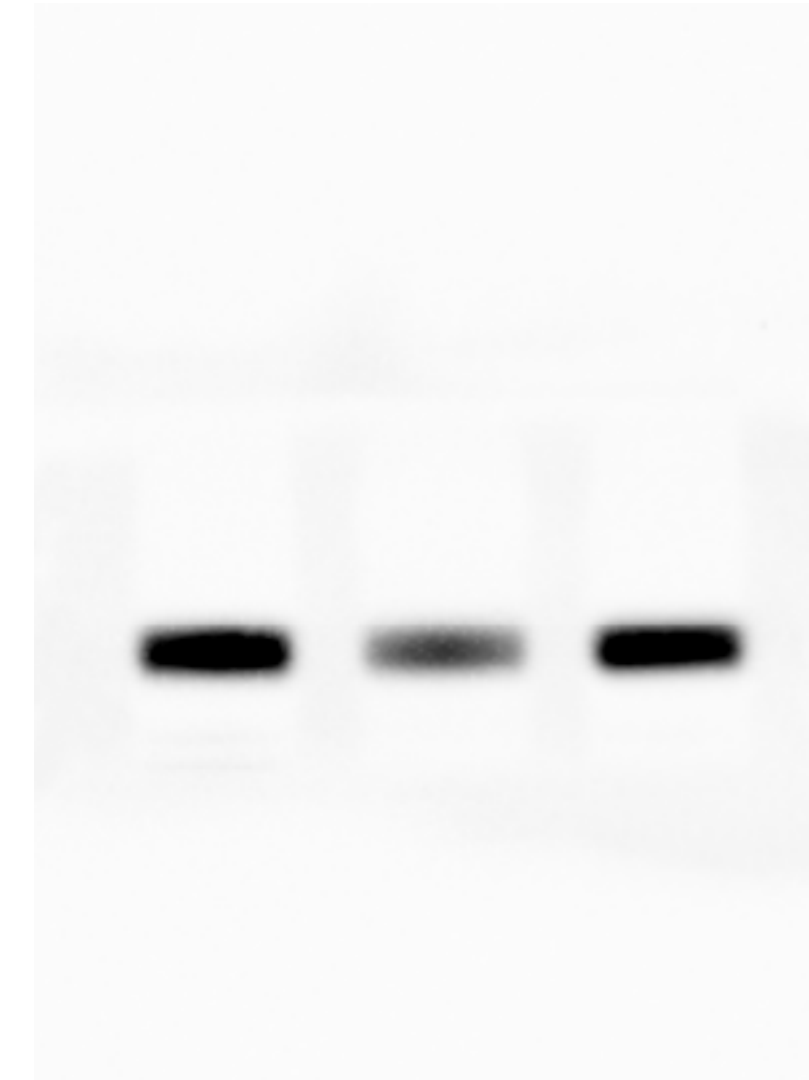

Supplement: Figure 7—source data 1. [file elife-81779-fig7-data1.pdf]
