# Supplementary material for: MCT1-dependent energetic failure and neuroinflammation underlie optic nerve degeneration in Wolfram syndrome mice
Source: eLife. 2023 Jan 16;12:e81779. doi: 10.7554/eLife.81779 (PMC9891717; doi:10.7554/eLife.81779)
Supplement: Supplementary file 2. [file elife-81779-supp2.docx]

| **Antigen** | **Company** | **Species** | **IF/IHC** | **WB** | **CO-IP** |
| --- | --- | --- | --- | --- | --- |
| β-ACTIN | Sigma-Aldrich A3853 | Mouse |  | 1:1000 |  |
| V5 | Thermo Fisher | Mouse | 1:500 | 1:1000 | 1:450 |
| RFP | MBL | Rabbit |  | 1:1000 | 1:500 |
| HA | Abcam (ab9110) | Mouse |  | 1:1000 |  |
| WFS1 | US bioscience | Mouse | 1:300 | 1:1000 |  |
| Calreticulin | Thermo Fisher | Rabbit |  | 1:1000 |  |
| Calnexin | Sigma-Aldrich C4731 | Rabbit |  | 1:2000 |  |
| GFP | ThermoFisher A10262 | Chicken | 1:1000 |  |  |
| GFP | Thermo Fisher A11120 | Mouse | 1:1000 |  | 1:450 |
| GFAP | Abcam ab4674 | Chicken | 1:1000 |  |  |
| GFAP | Millipore MAB360 | Rabbit | 1:1000 | 1:1000 |  |
| MCT1 | Thermo Fisher 76687 | Rabbit |  | 1:500 |  |
| MBP | Millipore MAB386 | Rat | 1:50 |  |  |
| GS | Millipore MAB302 | Mouse | 1:1000 |  |  |
