# Supplementary material for: MCT1-dependent energetic failure and neuroinflammation underlie optic nerve degeneration in Wolfram syndrome mice
Source: eLife. 2023 Jan 16;12:e81779. doi: 10.7554/eLife.81779 (PMC9891717; doi:10.7554/eLife.81779)
Supplement: Supplementary file 3. [file elife-81779-supp3.docx]

**List of the primers used in this study:**

| **Gene** |  | **Sequence (5' -> 3')** |
| --- | --- | --- |
| GFAP | F | CTCGGCCAGGTGAAGCATCA |
|  | R | CCTTCGGTTGGGGTAAAATGC |
| BDNF | F | CGGGGGTTGTTTGGACACCA |
|  | R | CCGACTTTTCCAGGGAAATTGGA |
| Glul | F | CCACACTAGGTGATGAATCCAGTGG |
|  | R | CCAGGACAATGCAGGTGAGAG |
| IL-18 | F | TCCTCTCCCCACAGATGTCCC |
|  | R | GCCGAGTCTTCAGCAGCAGC |
| C3b | F | TTCTGCCCCTCAGTACACAGC |
|  | R | CTTGCTGTGGAGGCTGAGGG |
| C4b | F | ATCACAGAACCTACAGTGAGTGTTG |
|  | R | CCAAGCGGTCCTTTGGTGTC |
| Lgals3 | F | CCCAGGAATGCATTCACAGAGG |
|  | R | CACAGAGCTGTGCATGGCAAT |
| Alpk1 | F | TGTTCCCGCCAGATACGTATCC |
|  | R | CCTCTCCAAATCCTGGGCCTT |
| Steap2 | F | TTCAGGTAACTCTGGCAGCCAATG |
|  | R | GAGCTCCTCTAAGCCTCGGCT |
| Uaca | F | CCTGCAGGGATCGCCTCC |
|  | R | CTGTCGATGGGTTTCTCAGTTTCA |
| Actin | F | CCATAGGTTTGTGGCACTGACAA |
|  | R | CCCCAAGCCCTCTCAGAGATGA |
| C1rl | F | GCAGGTCCTGGCCCTGTACA |
|  | R | CCTCAGCTGCACTTGCAGGAG |
| C1s | F | GGTGTACCCTCAGGTGTGCAA |
|  | R | CCAGCCATCCAGGTCTGTGTC |
| Vimentin | F | GGGCTCTTCTTCCAGGTTATGATG |
|  | R | CCCAGTAGGACTGGGTGACTT |
| VEGF | F | GGCGTTGCATCCAGGTTTTGA |
|  | R | CCGTGGTGGAGTTCACCACCTT |
| FGF2 | F | CTGCCTTCGACCGCTTACG |
|  | R | CAGAGCCATGATGTAGCTCAG |
| Lcn2 | F | CAAGCAGCGACGCATCAAG |
|  | R | GGGTTTGAGCGCGATCATATT |
| Wfs1 | F | GGGCAAGAGCCATCCTTTCAA |
|  | R | CTGTTCATCGTGTGGTACGTG |
| Edn2 | F | CGACGCCACCTACCATACC |
|  | R | CGGGCATCACCGAGTGTTC |
| Agtr1a | F | ATTCAACGAAGCCCACTACCCAGA |
|  | R | ATCCTTGGCTGACTTGAGGATGGA |
| 18S | F | GGTGAAATTCTTGGACCGGC |
|  | R | GACTTTGGTTTCCCGGAAGC |
